# Social Media and Chronic Pain: What Do Patients Discuss?

**DOI:** 10.3390/jpm12050797

**Published:** 2022-05-14

**Authors:** Lisa Goudman, Ann De Smedt, Maarten Moens

**Affiliations:** 1 Department of Neurosurgery, Universitair Ziekenhuis Brussel, Laarbeeklaan 101, 1900 Brussels, Belgium; maarten.moens@uzbrussel.be; 2 STIMULUS Research Group (reSearch and TeachIng neuroModULation Uz bruSsel), Vrije Universiteit Brussel, Laarbeeklaan 103, 1900 Brussels, Belgium; ann.desmedt@uzbrussel.be; 3 Center for Neurosciences (C4N), Vrije Universiteit Brussel, Laarbeeklaan 103, 1900 Brussels, Belgium; 4 Pain in Motion (PAIN) Research Group, Department of Physiotherapy, Human Physiology and Anatomy, Faculty of Physical Education and Physiotherapy, Vrije Universiteit Brussel, Laarbeeklaan 103, 1090 Brussels, Belgium; 5 Research Foundation—Flanders (FWO), 1090 Brussels, Belgium; 6 Department of Physical Medicine and Rehabilitation, Universitair Ziekenhuis Brussel, Laarbeeklaan 101, 1900 Brussels, Belgium; 7 Department of Radiology, Universitair Ziekenhuis Brussel, Laarbeeklaan 101, 1090 Brussels, Belgium

**Keywords:** chronic pain, patient opinion, social media communities, qualitative analysis, natural language processing

## Abstract

A high number of online support groups have been created on social media platforms to reinforce personal empowerment and social support. The goal of this study was to perform natural language processing by constructing a bag-of-words model and conducting topic modelling based on posts extracted from a chronic pain community. The subreddit called ‘r/sChronicPain’ was used to investigate communication on social media platforms for chronic pain patients. After data cleaning and lemmatisation, a word cloud was constructed, and the most frequent words and most frequent body regions were counted. Latent Dirichlet allocation was used to perform topic modelling. In the final analysis set, 937 unique posts were included. The most frequent word was ‘pain’, followed by ‘doctor’, ‘day’, ‘feel’, ‘back’, ‘year’, and ‘time’. Concerning the body regions, ‘back’ was most often mentioned, followed by ‘neck’ and ‘leg’. Based on coherence scores, one topic was extracted with ‘pain’ as the keyword with the highest weight. In line with the allocation of chronic low-back pain as a major health problem and increasing prevalence, back pain was most often mentioned. It seems that the primarily treatment trajectories that are proposed by medical physicians are discussed on social media, compared to interventions by other healthcare providers.

## 1. Introduction

Online support groups are designed to reinforce general well-being, a sense of control, self-confidence, feelings of more independence, and social interactions [1]. As such, they can foster personal empowerment for people in distress and are known to provide social support [1,2]. Within patients with chronic pain, defined as pain that lasts or recurs for more than three months [3], associations with stress are already demonstrated [4], and more specifically with a high prevalence of psychological distress [5]. One’s position in the social hierarchy, i.e., the social gradient of health, is a major determinant of health, which influences the development and evolution of chronic diseases [6]. Job dissatisfaction, low job control, minimal social support, depression, and interpersonal conflict are associated with, for example, the incidence of low-back pain [7]. Despite the reports in literature that social factors have been shown to affect chronic pain diseases [8,9], they often remain undocumented [10]. In 1987, Gil et al. revealed that pain behaviour varied as a function of the level of satisfaction with social support [11]. Nowadays, it is not surprising that several authors highlighted the potential value of social media and online support groups in providing social support in the setting of chronic pain [12,13].

Reddit is one of the largest content-sharing social media platforms with >430 million subscribers [14] and 330 million monthly active users. It is a publicly available social networking site that allows individuals to create posts within a variety of communities (called subreddits) such as science, sports, or politics. The idea is that users can comment and vote on the posts of other users, leading to the opportunity to have long conversations over time, group discussions and dissemination of information and personal opinions [15]. Reddit allows users to post text comments, but also enables users to post images or links. Depending on the number of votes, posts can gain more or less visibility. Thus, Reddit is a useful platform for real-time insights into the patterns, opinions, and experiences of particular groups and populations [16].

Nowadays, social media platforms are actively used to disseminate information to the public at large, and medical professionals display active participation in them [17]. The use of social media to spread surveys is generally accepted in healthcare [18]. In recent years, Reddit has been used in several studies on substance use and addiction and how social support and networking can help users to quit cannabis use [15,19]. Nevertheless, up till now, few researchers have used social media channels to evaluate information circulating on social platforms related to patients with chronic pain. Therefore, the goal of this study was to perform natural language processing by constructing a bag-of-words model and conducting topic modelling based on posts extracted from a subreddit about chronic pain.

## 2. Materials and Methods

### 2.1. Data Collection

The subreddit called ‘r/ChronicPain’ was used to retrieve posts of chronic pain patients. This community was created on 3 December 2009 and currently has 56,100 group members, according to the information on the webpage. Data for text mining were extracted on 6 September 2021 using Python Reddit API Wrapper (PRAW). The 1000 most popular hot, top, new, and rising posts (based on a sorting algorithm implemented within Reddit (Reddit Inc., San Francisco, CA, USA)) were extracted from the subreddit. Post identification, author, number of comments, score, created time, url, and the content of the post were retrieved for each post. The four datasets were merged whereby only unique posts were withheld for data analysis. 

The study protocol was approved by the ethics committee of Universitair Ziekenhuis Brussel (B.U.N. 1432021000559). A consent to participate was not required for this study. All information collected from this study was from the public domain, and no interactions took place with the users. Personally identifiable information was removed from the study results.

### 2.2. Data Preparation

Data cleaning consisted of removing stop words, removing hyperlinks, emoji, and punctuations and converting the text to lowercase. To remove stop words, the commonly used stop words in English as implemented in the Natural Language Toolkit (NLTK) were combined with a manual addition of stop words that were present in the extracted text. Afterwards, lemmatisation (the process of combining the inflected forms of a word in order to work with the base form (root) of a word [20]) was performed. 

### 2.3. Word Cloud and Bag-of-Words

First, a word cloud was generated using the *wordcloud* package to obtain a general idea of the most frequently used words in the posts. Afterwards, a bag-of-words (i.e., a representation of text that describes the occurrence of words within the posts, without taking into account the order or structure of words in the posts) was calculated. Additionally, body regions, types of healthcare providers, and alternative therapies were recognised in the posts, and the appearance of each word was counted. The lists for each category were manually constructed by the authors.

### 2.4. Topic Modelling 

Latent Dirichlet allocation (LDA) is one of the most popular methods in topic modelling and can be considered an unsupervised generative probabilistic method for modelling a corpus [21]. LDA represents topics by word probabilities whereby the words with the highest probabilities in each topic usually give a proper representation of the idea of each topic [21]. The LDA model gives the topic of each post with a distribution over the topics, and each topic is a multinomial distribution over words in the corpus, thereby relying on a three-stage Bayesian probabilistic model [22]. In other words, LDA is a mathematical method for finding the mixture of words associated with each topic while simultaneously also determining the mixture of topics that describes each post [23]. Topic modelling was conducted using *NLTK* and *Gensim.* LDA can output as many topics as requested, whereby a low number of topics can result in too few or very broad topics, while a higher number of topics can result in topics that ideally should have been merged [24]. Therefore, the optimal number of topics was evaluated with topic coherence scores (i.e., topic coherence scores a single topic by measuring the degree of semantic similarity between high scoring words in the topic [25]) and more specifically the C_v_ measure [26]. This measure takes into account segmentation of the data into word pairs, calculation of word or word pair probabilities, calculation of a confirmation measure that quantifies how strongly a word set supports another word set, and aggregation of individual confirmation measures into the overall coherence score [24]. The number of keywords per topic was 10, as commonly used [24]. LDA was computed based on 1000 iterations. 

## 3. Results

The original dataset consisted of 4000 posts that were retrieved from ‘r/ChronicPain’. After deduplication, the dataset was reduced to 1948 unique posts (based on the url address of the post). Afterwards, posts that only contained a hyperlink or image without a body text were excluded, resulting in a final set of 937 unique posts that were included in the analysis. Those posts were written by 709 unique authors. 

### 3.1. Characteristics of the Posts

**Figure 1:**
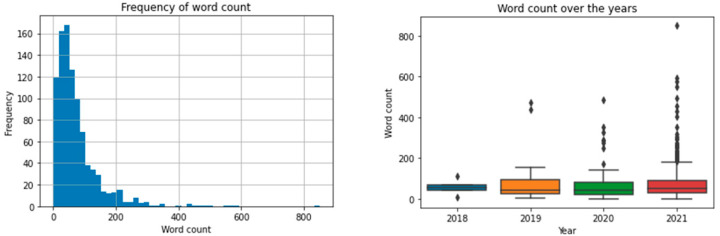
Histogram presenting the frequency of word counts per post (**left**). Word count was subdivided over the year in which a post was written to evaluate the trend in word count over years (**right**).

The posts had a median length of 53 (Q1–Q3: 28–90) words (Figure 1). The oldest post was written on 11 May 2018, while the most recent post was written on 6 September 2021. Overall, from 2018 to 2021, the length of posts seemed rather stable; however, more outlying observations with a higher word count were detected in the posts between 2020 and 2021 (Figure 1).

### 3.2. Bag-of-Words 

**Figure 2:**
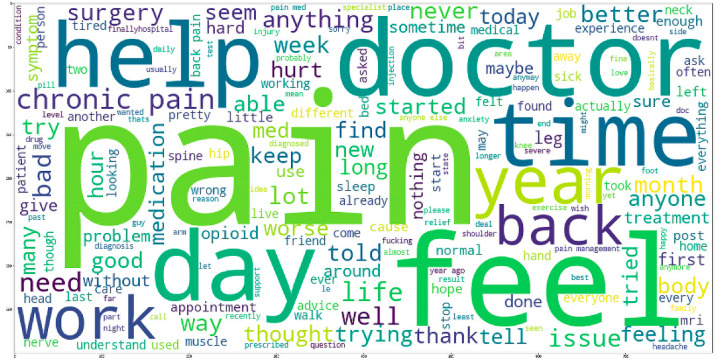
Word cloud of posts on the subreddit called r/ChronicPain.

Figure 2 presents a word cloud in which the most commonly occurring words across all posts are visualised. Common words such as stop words and prepositions were removed. Based on the bag-of-words approach, 7518 unique words were used in all posts. The most frequent word that was mentioned was ‘pain’ with a frequency of 2483, followed by ‘doctor’ (624 counts), ‘day’ (619 counts), ‘feel’ (609 counts), ‘back’ (557 counts), ‘year’ (540 counts), and ‘time’ (522 counts).

### 3.3. Frequency of Mentioned Body Parts

**Figure 3:**
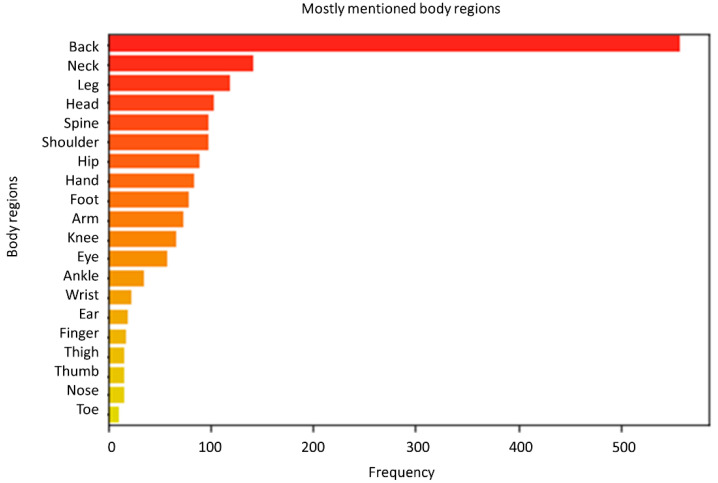
Bar plot presenting the frequency with which body regions were mentioned in posts.

The frequency of mentioning certain body parts was counted over all posts. The body region with the highest frequency was ‘back’ (557 counts), outperforming the frequency of all other regions. Other body regions with a high frequency were ‘neck’ (142 counts), ‘leg’ (119 counts), ‘head’ (103 counts), and ‘spine’ (99 counts). A bar plot with the mentioned frequencies of body regions is presented in Figure 3.

Concerning the type of healthcare provider that was mentioned, ‘doctor’ outranked all other healthcare providers with 624 counts. This was followed by therapist (without further specification) with 35 counts, nurse with 34 counts, surgeon with 33 counts, and physiotherapist/physio with 23 counts. Other healthcare providers that were less frequently stated were: physician (13 counts (could be added to doctor)), psychologist (6 counts), and occupational therapist (2 times). With respect to alternative therapies, massage was mentioned 26 times, yoga 23 times, diet 12 times, meditation 7 times, mindfulness 5 times, chiropractic 3 times, cupping 3 times, relaxation 2 times, and reiki 1 time. Medication was mentioned 175 times, drug 91 times, opioids 78 times, and painkiller 18 times.

### 3.4. Topic Modelling

**Figure 4:**
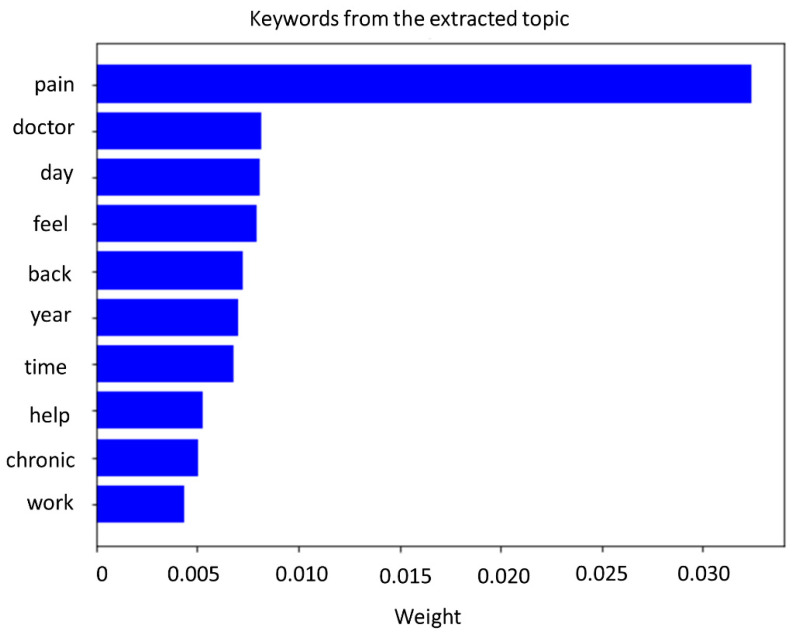
Keywords with corresponding weights that contribute to the selected topic, based on topic modelling with latent Dirichlet allocation.

Based on the coherence scores obtained after LDA, only one topic was revealed in the dataset. A topic is a combination of keywords with a certain weight to denote the contribution of a keyword to the topic. The following weights were found for the extracted topic: 0.032*“pain” + 0.008*“doctor” + 0.008*“feel” + 0.008*“day” + 0.007*“year” + 0.007*“back” + 0.007*“time” + 0.005*“chronic” + 0.005*“help” + 0.004*“work”. A visualisation of the weights of each keyword within this topic is presented in Figure 4.

## 4. Discussion

The aim of this study was to explore the content of posts that were written by patients with chronic pain on a popular social media platform, Reddit. A dominance of the word ‘pain’ was found in all analysis, which was not surprising in this community. Previous studies towards patient expectations and patient goalsetting in this population also denoted the importance of obtaining pain relief as an important factor [27,28,29]. Based on data from the European Social Survey in 2014, the prevalence of back/neck pain amongst men and woman at a pan-European level was 40%, followed by hand/arm pain at 22%, and foot/leg pain with a prevalence of 21% [30]. The prevalence of chronic low-back pain is estimated at 4.2% in individuals aged between 24 and 39 years old and 19.6% in those aged between 20 and 59 [31]. In a population-based survey, the prevalence of chronic low-back pain was estimated at 27.18% and chronic neck pain at 15.34% [32]. In line with the high prevalence of chronic pain in the lower back in the literature compared to other body locations, the number of patients that created posts relating to back pain also outnumbered the number of posts concerning other body regions.

Topic modelling only revealed one single topic, which seems to indicate healthcare seeking for pain. Surprisingly, there was no reference to family members, friends, or any other form of social support in the keywords, whereas receiving social support is probably the main reason for creating posts on this community. A review on informal pain-related social support indicated that in the majority of the included studies, the main focus was on spousal or partner social support with little information on the role of other sources of informal support such, as family members, friends, or neighbours [33]. Within the constructed word cloud, the words ‘family’ and ‘friend’ were mentioned 87 and 93 times, respectively, indicating a rather low ranking. A potential explanation for the lack of types of informal support in the topic modelling could be found in the type of data that were extracted from Reddit. A scoping review of YouTube to identify how adolescents with chronic pain use this social media platform revealed that multidisciplinary treatment options, alternative treatments, and the impact of pain on daily life comprised the main proportion of video content [34]. Comments on these videos were targeted at providing and receiving support, sharing suffering, and revealing the impact of pain on relationships and daily life [34]. It might be possible that more in-depth information regarding informal social support is not present in the posts but can be extracted based on comments that people are writing on submitted posts.

Currently, a multi- or interdisciplinary pain management program, relying on a biopsychosocial approach, is still considered one of the most appropriate treatment options for patients with chronic pain [35]. Multidisciplinary pain centre assessments typically rely on the input of several healthcare professionals, including pain medicine physicians, psychiatrists, nurses, psychologists, occupational therapists, and physiotherapists [36]. Apparently, on social media, medical physicians (doctors) are most often mentioned in posts (624 counts), presumably due to the proposal of treatment options and medication use since medication (abbreviated as med), surgery, and injection are mentioned as well in the word cloud, all related to the profession of medical physicians in the multidisciplinary teams. This could indicate that patients have a need to post the proposed treatment by their physicians to receive input from peers on, for example, alternative treatment options or concerns about medication. As such, active participation in these online support groups and communities could help patients to fully support the proposed treatment option and to contribute to empowerment of patients [37]. A growing minority of physicians themselves are active on social media to communicate directly with patients to augment clinical care [38]. Furthermore, it is thought that many users with medical professional backgrounds are active on Reddit; however, due to the lack of oversight from recognised health authorities, this is only an assumption [17]. Healthcare professionals could reinforce these effects by informing patients about the existence of support groups or communities that allow them to get in contact with peers, in case patients are not aware of these initiatives [37]. As such, healthcare professionals who are confronted with the care of chronic pain patients could have a two-fold function related to social media: (1) by providing links to websites with correct medical information in online support groups and (2) informing patients about the existence of online patient support groups. The intention is not that healthcare providers diagnose patients and write individual prescriptions on these support groups but rather take on the role of a moderator to provide patients with correct information. As such, a bidirectional information forum could be created, with input from physicians as well as peers (i.e., other chronic pain patients).

One of the main limitations of this study is that most Reddit app users are young, whereby users in their twenties and thirties account for 28.1% and 26.1% of the active users in the United States, respectively [39]. Therefore, the current findings might not be generalisable to the full adult population. Additionally, future studies should include comments raised on posts as well evaluate whether other patterns become visible when exploring the interactions between people. Within the topic modelling, only one topic was revealed, which was described as healthcare seeking for pain. Nevertheless, this is only the label that was placed on this topic, whereby other labels could be used to describe the 10 words within this topic as well.

## 5. Conclusions

In line with the high prevalence of chronic low-back pain in the literature and the finding that most chronic pain patients still have a primary goal to obtain pain relief, both concepts were also found in the analysis of written posts of chronic pain patients on social media with ‘pain’ as the most frequently reported word and ‘back pain’ as the most frequent body location. Based on natural language processing, it might be suggested that treatment trajectories proposed by medical physicians are most often discussed on the subreddit for chronic pain patients, compared to interventions by other healthcare providers.

## Data Availability

The data presented in this study are available on motivated request from the corresponding author.
